# Review on Applications of ^17^O in Hydrological Cycle

**DOI:** 10.3390/molecules26154468

**Published:** 2021-07-24

**Authors:** Yalalt Nyamgerel, Yeongcheol Han, Minji Kim, Dongchan Koh, Jeonghoon Lee

**Affiliations:** 1 Department of Science Education (Earth Sciences), Ewha Womans University, Seoul 03760, Korea; yalaltn@gmail.com (Y.N.); minji.jane.kim@gmail.com (M.K.); 2 Korea Polar Research Institute, Incheon 21990, Korea; yhan@kopri.re.kr; 3 Korea Institute of Geoscience and Mineral Resources, Daejeon 34132, Korea; chankoh@kigam.re.kr

**Keywords:** ^17^O-excess, kinetic fractionation, stable water isotopes

## Abstract

The triple oxygen isotopes (^16^O, ^17^O, and ^18^O) are very useful in hydrological and climatological studies because of their sensitivity to environmental conditions. This review presents an overview of the published literature on the potential applications of ^17^O in hydrological studies. Dual-inlet isotope ratio mass spectrometry and laser absorption spectroscopy have been used to measure ^17^O, which provides information on atmospheric conditions at the moisture source and isotopic fractionations during transport and deposition processes. The variations of δ^17^O from the developed global meteoric water line, with a slope of 0.528, indicate the importance of regional or local effects on the ^17^O distribution. In polar regions, factors such as the supersaturation effect, intrusion of stratospheric vapor, post-depositional processes (local moisture recycling through sublimation), regional circulation patterns, sea ice concentration and local meteorological conditions determine the distribution of ^17^O-excess. Numerous studies have used these isotopes to detect the changes in the moisture source, mixing of different water vapor, evaporative loss in dry regions, re-evaporation of rain drops during warm precipitation and convective storms in low and mid-latitude waters. Owing to the large variation of the spatial scale of hydrological processes with their extent (i.e., whether the processes are local or regional), more studies based on isotopic composition of surface and subsurface water, convective precipitation, and water vapor, are required. In particular, in situ measurements are important for accurate simulations of atmospheric hydrological cycles by isotope-enabled general circulation models.

## 1. Introduction

Two elements-oxygen and hydrogen, in water molecule with their stable isotopes (^16^O, ^17^O, ^18^O, ^1^H, and ^2^H or D) and their ratios (most commonly D/H or ^18^O/^16^O) have been widely used as hydrological or climatological tracers because of their sensitivity to environmental conditions during the evaporation, condensation, melting, and freezing of water [1,2,3,4,5,6]. Generally, lighter isotopes (^1^H, ^16^O, and ^17^O) are more likely to evaporate into the gas phase, while heavier isotopes (D and ^18^O) tend to condense into the liquid or solid phase. Furthermore, the oxygen (expressed as δ^18^O) and hydrogen (expressed as δD) isotopic ratios in precipitation are linearly correlated and this relationship is expressed by the Global Meteoric Water line (GMWL) [7]. Changes in the isotopic compositions generally represent the dependence of spatial factors (latitudinal, continental, altitude, and seasonal effects), and deviations from the GMWL at a specific location indicate the climate and environmental conditions [2,8].

Isotopic values have been extensively used in many studies to investigate moisture transport, air mass circulation, precipitation patterns [3,9,10,11], snow hydrology, water movement and balance [12,13,14,15] and the atmospheric water cycle through simulations based on isotope-enabled models [16]. The most depleted isotopic values are observed in polar precipitation, which is formed from cold air masses, and seasonal high and low values are observed, especially in coastal regions [2,4,5]. Thus, isotopic records of polar precipitation (accumulated layers of snow) are used for ice core age dating and as an important indicator of past climate and environmental changes [4,5,7,16,17,18,19,20]. Moreover, in temperate regions, isotopic signals in permafrost (ice wedges) are considered as a paleoclimate tracer [21,22].

In the triple oxygen isotope system, ^17^O is the one of the naturally occurring stable isotopes of oxygen with the lowest abundance (0.038%) [23,24], and the enrichment of H_2_^17^O in water is about half that of H_2_^18^O [1]. With the growing interest and recent developments in analytical instruments, it has become possible to measure small variations of ^17^O in meteoric waters, terrestrial rocks, and minerals [25,26,27]. A mass-dependent between the ratio of ^17^O/^16^O and ^18^O/^16^O is less sensitive to temperature, and hence, it is a useful indicator of the kinetic fractionation process. Many studies have used ^17^O as a proxy for certain conditions (relative humidity and wind speed) in the moisture source region [10,11]. The relative abundance of ^17^O in meteoric water is preferentially reported in terms of ^17^O-excess [^17^O-excess = ln(δ^17^O + 1) − 0.528×ln(δ^18^O + 1)][26]. ^17^O-excess is an effective parameter for investigating the global hydrological cycle, especially for studying the changes in the relative humidity in the oceanic source region and diffusion-induced kinetic fractionation process [9,10]. Compared with the relative excess of deuterium, which is termed d-excess (d-excess = δD − 8 × δ^18^O) and which is widely used as an indicator of kinetic fractionation processes [28,29,30,31,32], ^17^O is less dependent on the temperature at evaporation and condensation sites [33]. Thus, the use of ^17^O-excess in combination with δ^18^O and d-excess provides a potential means to unravel the effect of kinetic fractionation during evaporation at the moisture source, along the transport path, and during the condensation of precipitation [8,10,28,33]. Moreover, supersaturation conditions and cloud ice crystal formation in polar regions are influential in the ^17^O composition [30,34].

Although there have been numerous studies on the use of this rare isotope for investigating the water cycle, the processes controlling the spatiotemporal distributions of ^17^O in the fundamental hydrological cycle are still not well understood [35,36] and more data is required, particularly on less evaporated water bodies (river, precipitation) [36,37]. Aron et al. [36] proposed the introduction of multiple meteoric water lines in the expression for the variation of ^17^O-excess to indicate the importance of regional or local effects on the ^17^O pattern. Tian et al. [37] also provided evidence that the ^17^O-excess distribution indicated local climatic and geographical conditions rather than general large-scale spatial patterns. Surma et al. [38] mentioned the need to broaden the spatial coverage and long-term observations for refining the global monitoring network and to enable better prediction by the general circulation model. In this review, we introduce the potential applications of ^17^O into the hydrological cycle by summarizing the published literature on ^1^^7^O-based studies of the hydrological cycle. This paper is organized as following. Section 2 introduces the general definition and isotopic fractionation of ^17^O and analytical methods (dual-inlet isotope ratio mass spectrometry and laser absorption spectroscopy) for measuring the ^17^O. Section 3 summarizes the published ^17^O isotopic values for different sample types and describes the main findings of previous studies. Finally, on the basis of the implications of previous studies, future research needs are highlighted.

## 2. ^17^O Isotope Measurement in Water

### 2.1. ^17^O Definition and Isotopic Fractionation

^17^O is the one of the naturally occurring stable isotopes of oxygen, and also it has the lowest abundance, 0.038% [23] (Table 1). The distribution of isotopes in hydrological systems is controlled by isotope fractionation processes. Oxygen isotopes fractionate through mass-dependent isotopic fractionation, which includes kinetic processes, isotopic exchange reactions, and physicochemical phenomena (diffusion, condensation, and evaporation) [1,28]. Moreover, non-mass-dependent fractionation can be induced by chemical effects [39].

Small variations in the ratio of a rare isotope to an abundant isotope, such as ^17^O/^16^O and ^18^O/^16^O, due to the mass-dependent fraction, are observed during equilibrium isotope exchange and diffusion of water vapor [1]. Isotopic fractionation is expressed in terms of the fractionation factor (α). For example, the fractionation factor between a liquid (l) and a vapor (v) is defined as

*α_l/v_ = (*O/^16^O)_l_/(*O/^16^O)_v_ (1)

where * denotes 17 (^17^O) or 18 (^18^O). Barkan and Luz [40] reported the liquid-vapor equilibrium fractionation factors for ^17^α_l/v_ and ^18^α_l/v_ to be 1.00496 and 1.00940 at 25.3 °C, respectively, and highlighted their insensitivity to temperature (in the range 11–42 °C). The fractionation factors of ^17^α_l/v_ and ^18^α_l/v_ are related by an exponent *θ* [40]:

^17^α_l/v_ = (^18^α_l/v_)*^θ^*  or   *θ* = (ln^17^α_l/v_)/(ln^18^α_l/v_) (2)

The liquid-vapor equilibrium fractionation exponent (*θ*_eq_) was first estimated to be 0.528 by Meijer and Li [41], and later, Barkan and Luz [40] obtained an estimate of 0.529 ± 0.001; it is independent of temperature [40]. Furthermore, the kinetic fractionation effect of the diffusion (*θ*_diff_) of water vapor through air was reported as 0.5184 ± 0.0003, with ^17^α_diff_ and ^18^α_diff_ being 1.0146 ± 0.0002 and 1.0283 ± 0.0003, respectively [29]. Later, ^18^α_diff_ (H_2_^18^O and H_2_^16^O diffusion in air above the ocean) was reported to be 1.008 by Uemura et al. [42]. However, for diffusive fractionation, a slight temperature dependence (0.00002/15 °C decreasing trend if the trend is linear) are considered and still uncertain [43]. On the basis of the small difference between *θ*_eq_ and *θ*_diff_, equilibrium and kinetic fractionation processes can be distinguished.

The isotopic composition is expressed using the delta notation (δ), which is the ratio of *O/^16^O in a sample to that in a standard material multiplied by 1000:

δ*O = (*O/^16^O)_sample/_((*O/^16^O)_VSMOW2_ − 1)*1000 (3)

where VSMOW2 (or VSMOW, which stands for Vienna Standard Mean Ocean Water) is the water standard prepared by the International Atomic Energy Agency (IAEA). Thus, a sample’s δ*O is the relative ratio of ^18^O/^16^O or ^17^O/^16^O in the sample to that in the VSMOW2 standard, and it is presented in units of permil (%). Moreover, Standard Light Antarctic Precipitation (SLAP2 or SLAP) and Greenland Ice Sheet Precipitation (GISP) are also used as calibration materials. Schoenemann et al. [44] proposed the following VSMOW-SLAP normalization, which improves the accuracy of measurements and universalizes the data expression obtained from different laboratories.

(4) $$\delta \delta^{17}O_{sample}^{normalized}=\delta^{17}O_{sample}^{measured}\;\times \;(\delta^{17}O_{SLAP2}^{assigned}/\delta^{17}O_{SLAP2}^{measured})$$

Here, $\delta^{17}O_{SLAP2}^{assigned}$ is −29.6986‰, the value defined by Schoenemann et al. [44] as an accepted value for the SLAP2 standard (Table 1) and have been commonly used [30,36,37,38,45,46,47].

The reference lines for the relationship between δ^18^O and δ^17^O are very useful for interpreting triple oxygen isotope data, and the deviation from the reference lines are expressed by parameter ^17^O-excess [26], which is also reported as multiple terms including ^17^O_excess_ [48]_,_ and Δ^17^O [49]. Throughout the manuscript, we used the term ^17^O-excess and it is expressed as [29]:

^17^O-excess = δ′^17^O − λ_ref_ δ′^18^O or ^17^O-excess = ln(δ^17^O + 1) − λ_ref_ ln(δ^18^O + 1) (5)

Here, λ_ref_ is the slope of the δ′^17^O–δ′^18^O line. ^17^O-excess indicates the relative excess of ^17^O in meteoric waters compared with ocean water. In the equation, δ′ is the logarithmic term of δ and is expressed as δ′ = ln(δ + 1); it linearizes the exponential relationship between isotopic compositions [50]. The parameter ^17^O-excess is very small, and it is multiplied by 10^6^ and expressed in units of per meg (1 per meg = 0.001%). The magnitude variation in the line δ′^17^O–δ′^18^O is very small (the difference between *θ*_eq_ and *θ*_diff_ corresponds to very small magnitudes of δ′^17^O and δ′^18^O, and it is commonly described by the ^17^O-excess-δ′^18^O line) [36]. ^17^O-excess represents the mass-dependent deviation from the reference relationship [51]. Luz and Barkan [26] reported the reference relationship as a meteoric water line that was plotted with a slope of 0.528. Thus, ^17^O-excess indicates the magnitude of deviation from this reference line.

### 2.2. ^17^O Measurement

An isotope ratio mass spectrometry (IRMS) with fluorination reaction, for converting water to O_2_ with CoF_3_, was developed by Baker et al. [52]. In the earliest studies, water was converted to O_2_ gas, based on electrolysis using CuSO_4_ [41] and fluorination using BrF_5_ [53]. Baker et al.’s [52] method was modified by Barkan and Luz [40], and it is commonly used for the measurement of water isotopes with high precision [26,42,46,49,54]. Affek et al. [55] modified and proposed an approach to measure oxygen isotopes of CO_2_ equilibrated with water, based on the isotopic exchange at steady state between O_2_ and CO_2_ [56]. Recently, laser absorption spectroscopy has been developed, and it allows less laborious, simultaneous and direct measurements of water isotopologues [36,57].

#### 2.2.1. Dual-Inlet Isotope Ratio Mass Spectrometry

Continuous flow and dual-inlet IRMS have been used for the measurement of δ^18^O and δ^17^O on the basis of different ion currents corresponding to different isotopologues of water. Meijer and Li [41] reported accuracies of 0.10% for δ^18^O and 0.07% for δ^17^O and obtained the value of λ_ref_ as 0.5281 + 0.0015. Barkan and Luz [40] modified the IRMS method with fluorination reaction (Equation 6), which was first developed by Baker et al. [52] and used in many studies.

2H_2_O + 4CoF_3_ = O_2_ + 4CoF_2_ +4HF (6)

According to the method described by Barkan and Luz [29], a water sample (2 µL) is injected into a heated nickel tube and converted by fluorination into O_2_ gas using CoF_3_ reagent. The O_2_ gas is then adsorbed on 5 Å molecular sieves contained in a coiled trap at −196 °C. He gas is used as a carrier gas, and after trapping the entire amount of O_2_ gas, the He flow is diverted and the residual He in the trap is pumped away. The gaseous O_2_ is then trapped in a stainless-steel manifold, which is immersed in a liquid helium tank. The manifold is connected to a dual-inlet mass spectrometer, and δ^17^O and δ^18^O are measured simultaneously. Sample processing, including the fluorination step (≈30 min) and IRMS measurement, takes around 2.2 h for each sample. The IRMS measurement involves three runs, with each run comprising 30 measurements. The three runs (*n* = 90) are averaged, and δ^17^O and δ^18^O are determined with analytical errors of 0.006% and 0.003%, respectively. Atmospheric O_2_ was measured for use as the working standard, with an accuracy of 0.005% for δ^17^O and 0.004% for δ^18^O [40]. The root mean square error of the isotope analysis, performed with a Nu Perspective IRMS instrument, was 0.3% for δ^17^O and 0.9% for δ^18^O [36]. Schoenemann et al. [44] reported isotopic measurements with a reproducibility of 0.002% for δ^17^O and 0.004% for δ^18^O for this fluorination method; they used Thermo Finnigan 253. The two-point VSMOV2-SLAP2 normalization and drift corrections are applied, refining the accuracy of the measurements [44]. Wassenaar et al. [58] suggested the importance of calibration by primary working standards which can be altered by improper storage and handling and importance of lab standards for frequent monitoring of the instrument.

#### 2.2.2. Laser Absorption Spectroscopy

Laser absorption spectroscopy can be used for the continuous and direct measurement of water vapor with high precision and accuracy, and cavity ring-down spectroscopy (CRDS) is used for measuring the rate of decay of a laser beam by the beam-absorbing water molecules [59]. The off-axis integrated cavity output spectroscopy (OA-ICOS) laser absorption spectrometer, developed by Los Gatos Research, is based on near-infrared tunable diode laser absorption, with the laser coupled off-axis to a high-finesse optical cavity [60].

**Figure 1:**
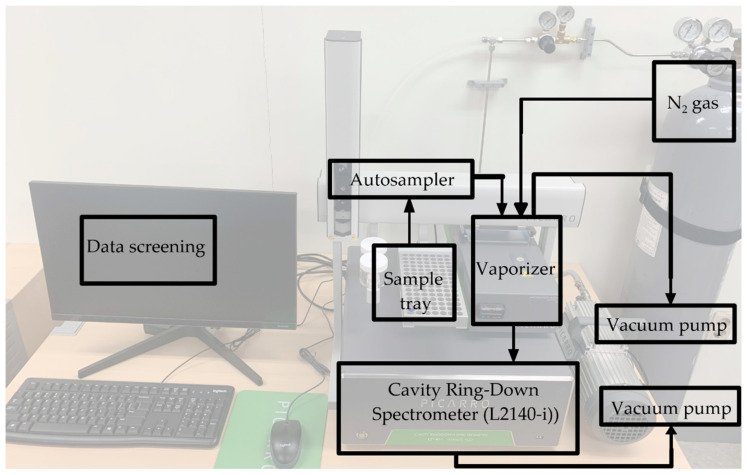
Schematic of the Picarro cavity ring-down spectrometer (L2140-*i*) installed at the Environmental Geochemistry Laboratory of Ewha Womans University.

Berman et al. [60] measured the GISP standard by OA-ICOS and reported an δ^17^O of −13.12 ± 0.05‰ and a ^17^O-excess of 23 ± 10 per meg; Steig et al. [61] reported a measurement with a precision below 8 per meg for δ^17^O by a modified version of CRDS (L2130-*i*-C by Picarro Inc., Santa Clara, CA, USA), which detects strong absorption of H_2_^17^O and H_2_^18^O in a wavelength region centered at 7193 cm^−1^. Currently, Picarro Inc.’s water isotope analyzer model L2140-*i* is commercially available for the measurement of δ^17^O. Liquid water samples (≈1.8 µL) are introduced into an automated vaporization system by an autosampler. Water samples evaporate and they are transported by a diaphragm vacuum pump into the optical cavity of L2140-i (Figure 1). The vapor pressure in the cavity is recommended to be in the range of 17,000–23,000 ppm by the manufacturer, because the isotopic ratios are strongly dependent on the water mixing ratio of the air [62]. The sample volume, sampling speed, measurement repetition, and sampling sequences can be controlled through the autosampler [59]. Sturm and Knohl [62] mentioned less fluctuated temperature conditions to prevent changes in cavity temperature. Pierchala et al. [59] reported the short-term precision of δ^17^O and ^17^O-excess as 0.028% and 11 per meg, respectively. A memory effect between consecutive samples and the selection of a calibration standard having isotopic values closest to those of the sample were identified as important factors for improving the precision of δ^17^O and ^17^O-excess [59]. For the improvement of the accuracy and precision of the measurement in laboratories, in-house control standards have been suggested [63]. Berman et al. [60] suggested the memory effect from mixing by syringe and water adhesion to internal surfaces of the instrument. Schauer et al. [57] suggested the syringe actuation which may weaken with large injection numbers, thus fewer injection numbers were recommended. Moreover, data-processing approaches for memory correction and sample sequence avoiding the large difference of isotopic values were also recommended. Salt liners (mesh inserts in the vaporizer unit) and salt-specific corrections are required if water samples contain high minerals and dissolved organic components. The use of a saline water sample could result in an incomplete evaporation of the sample [64]. In laser absorption instruments, organic compounds (ex. methanol) affect spectral interference and thereby cause the isotopic values to diverge from the actual values [65].

## 3. Use in Hydrological Studies

Small variations in the ^17^O/^18^O relationship can result from mass-dependent equilibrium (fractionation caused by different saturation vapor pressures) and kinetic fractionation processes (fractionation induced by different diffusivities of water vapor isotopologues) [33]. The parameter ^17^O-excess provides information about moisture transport and conditions (relative humidity and wind speed) in the moisture source region [7,35], and it is known to be primarily sensitive to relative humidity and less sensitive to temperature and Rayleigh distillation during moisture transport and precipitation. There are several controlling factors for the ^17^O-excess pattern, including the moisture source, mixing of water vapor, rain re-evaporation (mostly in low and mid-latitudes) and supersaturation (at low condensation temperatures) [66]. Although the ^17^O-excess in precipitation is primarily interpreted as indicating variations in the relative humidity in the moisture source regions, precipitation in polar and dry regions suggests the importance of fractionation by snow formation and raindrop re-evaporation processes.

Pioneering measurements of δ^17^O and δ^18^O were performed by Barkan and Luz [40] by using two international water isotope standards, SLAP and VSMOW. Later, the relationship between δ′^17^O and δ′^18^O was obtained by Luz and Barkan [26] as the GMWL (Equation 7 below). This line is based on the measurements of the GISP, SLAP, polar snow samples [28], and a set of meteoric waters, including precipitation, surface water, cave water, and snow, mostly from Europe and Asia.

δ′^17^O = 0.528 * δ′^18^O + 0.033   or   ln(δ^17^O + 1) = 0.528 * ln(δ^18^O + 1) + 0.033 (7)

However, freshwater samples are not covered in this meteoric water line, and the line has been recently refined and discussed by Aron et al. [36]. The observed slopes (λ_obs_) in the study of Aron et al. [36] appeared to decrease in the order of snow and ice (0.5285) > ocean (0.5278) > precipitation (0.5273) > surface and subsurface water (0.5261) > plant water (0.5188). This indicates the dominance of kinetic processes at low slopes (close to *θ*_diff_ = 0.5184 ± 0.0003) and equilibrium fractionation at higher slopes (close to *θ*_eq_ = 0.529 ± 0.001).

**Figure 2:**
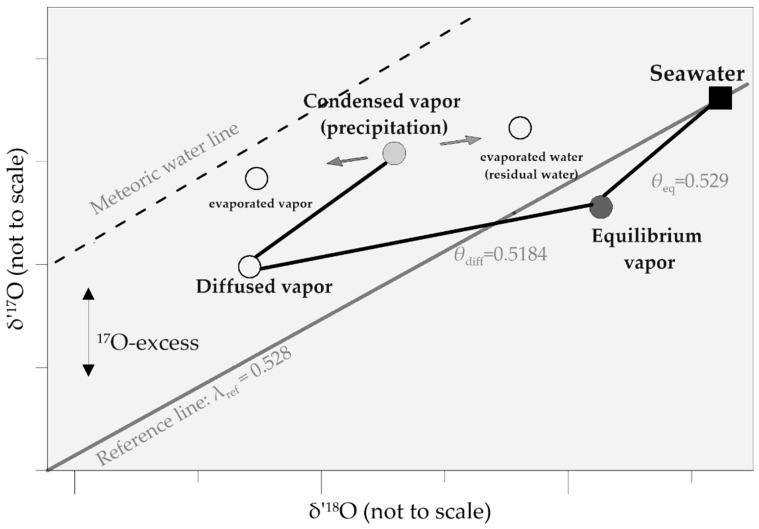
Schematic of δ′^17^O and δ′^18^O fractionation in the hydrological cycle. This figure is a simplified modification of the descriptions from Luz and Barkan [26] and Aron et al. [36].

For water vapor, ^17^O-excess increases with kinetic fractionation [28]. Kinetic fractionation during the condensation of water vapor to ice in high-latitude precipitation can also be described by the ^17^O-excess together with the d-excess [33]. A schematic of δ′^17^O and δ′^18^O behavior is shown in Figure 2. Evaporated vapor from seawater diffuses through an unsaturated atmosphere, increasing the partition of ^17^O. ^17^O-excess decrease in condensed vapor (precipitation). Post-condensation evaporation will increase ^17^O-excess in evaporated vapor and decrease ^17^O-excess in residual evaporated water.

Average ^17^O-excess values have been reported in various ranges from −144.4 per meg (Pond in Iran) to 49.7 per meg (Firn core, Greenland) (Table 2). Evaporated water samples (e.g., leaf water, pond, and lake) show negative ^17^O-excess values, indicating the presence of kinetic effects [36]. While tap water and precipitation values show positive ^17^O-excess, snow and ice core samples show higher positive values.

However, the spatial pattern is not globally consistent due to the lack of sufficient observations, as well as large variations in the local-to-regional scale [36]. Globally, δ′^17^O and δ′^18^O values do not conform to a single meteoric water line, indicating the existence of large spatial differences (regional hydrological processes) in the samples included [36]. Moreover, biases have been suggested on the basis of the water type, sampling bias, and sampling duration. Aron et al. [36] suggested that the δ′^17^O-δ′^18^O relationship should be refined by using unevaporated waters (flowing surface water and monthly precipitation data). Meteoric water’s ^17^O-excess shows significant variation in the compilation of Aron et al. [36], and a single line cannot express the variation.

### 3.1. Low- and Mid-Latitude Hydrology

Meteoric waters are generally in agreement with the slope of 0.528 [39,67], which is close to the equilibrium fractionation exponent (*θ*_eq_ = 0.529) [40]. Re-evaporation of rain drops during warm precipitation and convective storms, and mixture of moisture are considered as the main processes behind the ^17^O-excess variation in low- and mid-latitude precipitation [66].

Marine water vapor (southern Indian and Southern Ocean), which can be assumed to be less affected by precipitation and snow formation processes, was first studied by Uemura et al. [42]. For marine vapor (15 m above ocean surface), ^17^O-excess ranged between −6 and 46 per meg (mean: −13.5 per meg), and it negatively correlated (slope: −0.64 per meg/%) with the normalized relative humidity (Rh_n_, which is the ratio of the water vapor concentration in air to the saturated vapor concentration at the ocean surface temperature). The average impact of the sea surface temperature (SST) on ^17^O-excess was insignificant, amounting to about 0.1 per meg/°C, and the observed positive correlation between the SST and ^17^O-excess was explained as a consequence of changes in relative humidity at the local scale. The study suggested a small contribution of the air mass mixing from high elevations into the atmospheric boundary layer to ^17^O-excess.

In this context, a study found that ^17^O-excess measurements in tap waters across continental US (mid-latitudes), which is a proxy for precipitation, ranged from −6 to +43 per meg, with a mean of 17 ± 11 per meg [46]. This study reported the importance of the effect of continental recycling of moisture on the ^17^O-excess distribution (large range of 40 per meg) rather than changes in relative humidity (low variation of <5%) at the ocean source. Furthermore, it was found that ^17^O-excess in precipitation can be lowered by re-evaporation and mixing processes, and the slopes ranged between 0.526 and 0.527, close to equilibrium fractionation (steady-state evaporation). It was also suggested that atmospheric turbulence and moisture mixing during convective processes (non-steady) should be considered in the interpretation of ^17^O-excess patterns. This study revealed the latitudinal difference in the distribution of ^17^O-excess, which has low values in southern locations because of the re-evaporation of precipitation and convective processes, and which disappears in the northern location (cold and snowy). This continental-scale data showing differences in local climate is very useful for global circulation models and hydrological studies that incorporate ^17^O-excess.

Tap water (as precipitation proxy) across China was studied by Tian et al. [37]. Most water samples indicated the equilibrium fractionation effect, except for the north-west region, where the kinetic effect dominates (re-evaporation effect). No significant seasonal dependence was observed in ^17^O-excess. Furthermore, this study mentioned that the ^17^O-excess distribution is more relevant to precipitation formation mechanisms under local climatic and geographical conditions compared with consistent large-scale spatial patterns.

The effect of extreme evaporation, which decreases ^17^O-excess in residual water, was studied by Surma et al. [54]. Natural waters (Helmand River, shallow wells, artificial freshwater reservoirs, irrigation channels from a delta plain, ephemeral terminal lakes, and residual ponds) from the arid and semiarid environment of the Sistan Oasis, Iran, were investigated in this study. The isolated (un-recharging water bodies) and heavily evaporated (progressive evaporation in this arid and semiarid environment) water bodies showed very low ^17^O-excess, reaching −167 per meg, and a distinct ^17^O-excess-δ′^18^O curve that indicated the region’s relative humidity.

To understand the evaporation dynamics from natural water samples, a study examined dew and precipitation samples from dry (Gobabeb, Namibia), Mediterranean (Nice, France), and humid continental climates (Indianapolis, IN, USA) [68]. This study confirmed that the low ^17^O-excess of dew in dry areas (Gobabeb and areas in Indianapolis with a temperature over 14.7 °C) was related to kinetic fractionation with evaporation, while the humid continental area (Nice, France) showed no significant evaporation effect. They also mentioned that the local relative humidity was important for the equilibrium or kinetic fractionation of dew formation.

Triple isotopic measurements in precipitation, drip water and speleothem fluid inclusions from Milandre Cave (Boncourt, North West Switzerland) have been found to be potentially useful for the reconstruction of paleotemperature and moisture variations, particularly for Western Central Europe [69] Both precipitation and drip water measurements were around 18 per meg, while the speleothem fluid inclusions showed ^17^O-excess values, similar to those of surface precipitation. This study proposed that the speleothem fluid inclusions may have recorded information about past moisture sources.

The ^17^O-excess in precipitation in a tropical region during convective activity was studied in Landais et al. [70]. It was agreeable that the rain re-evaporation process explained the increase in ^17^O-excess. However, other factors (changes in the evaporative source, convection, and recycling along trajectories) also influenced convective precipitation. This study highlighted the need to focus on water vapor isotopic composition, isotopic fractionation associated with rain re-evaporation and the re-evaporation rate, and large-scale controls on ^17^O-excess variation in future research.

In a tropical region (Mpala in Kenya), Li et al. [47] found that the variation of ^17^O-excess in leaf water correlated with the relative humidity. This study mentioned the strong influence of the isotopic composition of ambient vapor with high relative humidity on the slope λ_transpiration_. Moreover, δ^17^O values in leaf water showed a very large variation, and this was likely to indicate the response of δ^17^O to the variation in the materials linked to leaf water (e.g., atmospheric CO_2_, plant tissue, biomaterials that record animal body water).

Two-year monitoring of ^17^O-excess in precipitation in a subtropical region (southern part of Okinawa Island, Japan) was reported in Uechi et al. [71]. For this location, the effect of diffusional fractionation during evaporation in the source region could be reconstructed from the ^17^O-excess in the precipitation. Thus, this study suggested that ^17^O-excess in precipitation in subtropical and tropical regions allows for the reconstruction of changes in the relative humidity in the moisture source region. Moreover, this study also emphasized that analyzing ^17^O in fluid inclusions in speleothem and calcite would be useful for understanding paleoclimate variations.

Surma et al. [38] studied atmospheric and snow from high altitude in Mt. Zugstpitze, Germany and indicated the dominate influence of synoptic processes (e.g., Rayleigh distillation and potential continental recycling) on the atmospheric vapor in mid-latitudes. This study also mentioned that there could be input from deep stratospheric intrusion in this location, however the potential influence was small due to the relatively high vapor content in the local vapor compared to the stratospheric vapor. Moreover, this study comprehensively synthesized and explained the distribution of ^17^O-excess and the crucial processes affecting it in more detail. The extension of spatiotemporal observations was noted as important for refining the global monitoring network and for better prediction by the general circulation model.

Recent measurements in stream waters across the Pacific Northwest in the US also show a link between relative humidity and ^17^O-excess. The effect of evaporation in the rain shadow of the Cascado Mountain on the ^17^O-excess of stream waters was large (higher ^17^O-excess corresponded to lower relative humidity) [72]. Later, Passey and Li [49] studied the evaporation effect on triple oxygen isotopes in a river, basin lake (terminal) and freshwater lakes by using an isotope mass balance model. A larger ^17^O-excess was observed in unevaporated water (river) compared with evaporated water (basin lake and freshwater lake). This study also reported that ^17^O-excess measurements in lacustrine carbonates were representative of precipitation and that their values were similar to those of catchment precipitation.

### 3.2. High-Latitude Hydrology

The influence of Rh_n_ changes in the moisture source region and a supersaturation-controlled kinetic isotope effect during in-cloud ice formation (below −20 °C) are mainly considered in ^17^O-excess variation of Antarctic precipitation and ice cores [73,74]. Snow formation (clear-sky precipitation or diamond dust) under supersaturation was likely to result in low ^17^O-excess in high latitudes and it differed from precipitation formed from cloud-derived and synoptic snowfall [28,30,34]. Moreover, changes in temperature and relative humidity at the source and site, changes in sea ice concentration and descending stratospheric water vapor influenced the ^17^O-excess variation [75]. Particularly, the stratospheric air inflow, which was mostly induced by polar vortex to polar troposphere, is more significant in high-latitudes than the low- and mid-latitudes where water content is high enough compared to the stratospheric inflow [38].

Pioneering research by Landais et al. [28] showed spatially (from coast to continent) constant ^17^O-excess values (~45 per meg) in surface snow from Antarctica, revealing the low sensitivity of ^17^O-excess to the temperature decreasing toward inland Antarctica. Moreover, the ^17^O-excess in a Vostok ice core was indicative of changes in oceanic conditions (humidity and wind speed) during the last 150,000 years. An increase in about 20 per meg was observed in ^17^O-excess, and it was related to the lower Rh_n_ and wind speeds in the interglacial period compared to the steady values in the glacial period. Although there could be intrusion from the recirculation of cold Antarctic air and vapor input from the tropical ocean, the surrounding oceanic vapor (source) was proposed as the major factor controlling the ^17^O-excess in Antarctic snow. This study suggested the future studies should combine d-excess with ^17^O-excess and vapor measurements for tropical areas.

However, the interglacial increase in ^17^O-excess corresponding to a lowering of Rh_n_ observed by Landais et al. [28] was debated in later studies. Winkler et al. [76] suggested that ^17^O-excess measurements in coastal sites are more reliable in reconstructing changes in normalized relative humidity compared with remote inland sites such as Vostok [28], where the local effects may be strong. They reported distinct ^17^O-excess values in Dome C and the Talos Dome site and suggested that the site conditions at each site (e.g., wind pattern), and different glacial-interglacial changes in the normalized relative humidity, were important.

Winkler et al. [76] studied ^17^O-excess changes from 1948 to 2008 in a snow pit near Vostok, by combining the model output from Laboratoire de Meteorologie Dynamique-Zoom (LMDZ) and a simpler Rayleigh-type model Mixed Cloud Isotopic Model (MCIM). This study suggested the complexity of influencing factors (in interannual scale) at sites, such as Vostok, with very low snow accumulation. Touzeau et al. [77] also mentioned that post-deposition effects are expected to have a significant effect on low-accumulation sites. The precipitation type was tropospheric snowfall, and the majority of precipitation events were a type of hoar-frost deposition and diamond dust; the surface humidity was very low in inland sites such as Vostok. Thus, although the water vapor amount in the stratosphere is low, it can change ^17^O-excess in remote inland sites.

The kinetic effect of strongly supersaturated conditions at low temperatures on the ^17^O-excess (^17^O-excess is positively correlated with δ^18^O) variation was reported for Dome C site in [77]. Furthermore, Miller [73] suggested the importance of the precipitation regime, whether diamond dust (decrease in ^17^O-excess or water vapor supersaturation) or cloud-derived precipitation (increase in ^17^O-excess), for the ^17^O-excess over the Antarctic ice sheet. Moreover, isotopic fractionation, which increased the ^17^O-excess values, was observed to result from moisture recycling processes (sublimation of surface snow) in summer precipitation over East Antarctica [78].

In contrast to the steady values toward the inland region in the study of Landais et al. [28], surface snow samples from Zhongshan station to the Dome A site showed a decrease in ^17^O-excess [45]. These lower ^17^O-excess values were explained by the kinetic fractionation induced by the supersaturation on ice crystals at low temperatures. This study suggested that differences in moisture sources and supersaturation conditions influenced the variability of ^17^O-excess and pointed out the need for more measurements from different sites in Antarctica.

On a broader scale, ^17^O-excess was reported to be low in the central East Antarctic Plateau and higher in the coastal marine-influence regions in Schoenemann et al. [30]. This work reported the spatial distribution of ^17^O-excess by combining the records for the West Antarctic Ice Sheet (WAIS) divide with those from Vostok [28], EPICA DOME C, and the TALDICE site [76]. Temporally, the variation of ^17^O-excess was large in inland sites over the last glaciation. These results remarkably highlighted the importance of the supersaturation of water vapor over ice, and also that ^17^O-excess was not solely dependent on changes in relative humidity over the moisture source region during glacial-interglacial variations. This indicates the effect of the temperature gradient in the moisture source, and at the precipitation site, on the ^17^O-excess distribution. The change in the boundary of the sea ice extent is another factor that should be considered when referring to the temperature gradient. The expansion of sea ice (or colder conditions) results in the supersaturation conditions appearing earlier, which would decrease ^17^O-excess. Moreover, the contribution of relatively enriched moist air evaporated from the sea surface would be restricted for a large sea ice extension. This study highlighted the possibility of reconstructing glacial-interglacial scale changes in climatic conditions (temperature and sea ice extent).

Risi et al. [66] simulated ^17^O-excess by using an isotope general circulation model and explained the main controlling processes. Convective processes and re-evaporation were important in the tropical region, while the effect of distillation, mixing of different air masses, and supersaturation were crucial at mid- and high-latitudes. The model requires the tuning of supersaturation, which is the main effect in Antarctica.

Schoenemann and Steig [75] simulated seasonal and spatial variations in ^17^O-excess and d-excess in Antarctic precipitation by using the Intermediate Circulation Model. A smaller variation (≈3 to 8 per meg) in seasonal ^17^O-excess was simulated over the Southern Ocean compared with a large variation of about 50 and 60 per meg for ice sheet and snowfall. Moreover, changes in the ocean surface relative humidity induced a small variation, the average variation being −1.3 per meg/%. This study suggested that the source region Rh_n_ and sea surface temperature partially contributed, while the effects from the surface temperature and water content of precipitable vapor were more important to the isotopic variability within the annual cycle.

The use of ^17^O-excess as a marker of the source of relative humidity was confirmed in water vapor, surface snow, and shallow ice core from the NEEM site, Greenland in Landais et al. [48]. The study in [43] experimentally reported and confirmed the relationship between the solid-vapor fractionation coefficients for δ^17^O and δ^18^O (0.528 *=* (ln^17^α_v/s_)/(ln^18^α_v/s_)). An increased ^17^O-excess was observed in the winter season, while lower values were observed in summer, indicating the seasonality of the air temperature and relative humidity in the evaporative source region.

Stratospheric air inflow, which has a high ^17^O-excess (of the order of 3000 per meg) [38,79] compared with the tropospheric vapor, causes a significant increase in ^17^O-excess in high-latitudes [28]. The contribution of evaporative conditions to ^17^O-excess is underestimated by the LMDZ model, suggesting that shortcomings in the simulation of ^17^O-excess by the GCM should be overcome [66].

## 4. Summary and Future Direction

With the recent analytical developments for measuring small variations in ^17^O in the triple oxygen isotope system, ^17^O has been used as a new tracer in studies on regional hydrology and surface hydrology, climate, and paleoclimatology. Dual-inlet isotope ratio mass spectrometry and laser absorption spectroscopy have been developed and commonly used to measure ^17^O. The reference relationship, with a slope of 0.528, was developed and, together with d-excess, it allows the separation of the effects of temperature and relative humidity in meteoric waters. ^17^O-excess is commonly used as a tracer for examining changes in relative humidity in the moisture source region, while the other influential processes are still being debated. While ^17^O-excess may be mainly influenced by the relative humidity in subtropical regions, in polar regions, especially in continental Antarctic sites, this influence is replaced by supersaturation effect, intrusion of stratospheric vapor input, post-depositional processes (local moisture recycling by sublimation), regional circulation patterns, sea ice concentration, and local meteorological conditions. At low and mid-latitudes, the ^17^O-excess pattern is mainly affected by changes in the moisture source, mixing of water vapor, re-evaporation of rain drops during warm precipitation, and convective storms. Thus, the spatiotemporal variation of ^17^O-excess is large and yet uncertain, indicating multiple meteoric water lines corresponding to the geographical location. Moreover, since the isotopic fractionation is complex in nature and since there is a lack of measurements compared to spatially large variations, more studies on isotopic composition of surface and subsurface samples, precipitation (convective), and water vapor are required. It is also necessary to provide isotope-enabled models with in situ measurements to perform accurate and robust simulations.

## Figures and Tables

**Table 1:** Oxygen isotopes, abundances (relative %), δ notation, and δ values of standard materials.

Isotope	Abundance	δ Notation	δ Value of VSMOW2	δ Value of SLAP2	δ Value of GISP
^16^O	99.76%	–			
^17^O	0.038%	δ^17^O	0	−29.6986‰ ^1^	−13.16 ± 0.05 ^1^
^18^O	0.20%	δ^18^O	0	−55.5‰ ^2^	−24.82 ± 0.08 ^1^

^1^ Schoenemann et al. [44]. ^2^ IAEA.

**Table 2:** Summary of published δ^18^O, λ_ref_ and ^17^O-excess in different samples. All values are averaged from published values. We note that the sampling location, period, and sampling numbers are distinct depending on the studies; thus, values are representative of local scale variations.

Sample Type	δ^18^O Range	^17^O-Excess Mean or Range (Per meg)	λ_ref_	Analytical Method	Time Scale	Reference
**Low- and mid-latitude Precipitation**						
Vapor (Mt.Zugspitze, Germany)	(−34.4 to –20.4)	(30 to 82)	0.5265	IRMS	2016 (Feb to May)	[38]
Cave water (Canada and USA)	−9.69 (−17.67 to −6.39)	0.05 (0.03 to 0.07)	0.530	IRMS	2005 (Feb, Jul)	[26]
Cave drip waters (Northwest Switzerland)	−8.7	19	–	Picarro CRDS	2010 (Nov) to 2014 (Jun)	[69]
Fluid inclusions (Northwest Switzerland)	−8.3	10		Picarro CRDS	2012 (Mar) to 2014 (Jun)	[69]
Leaf water (Mpala central Kenya)	7.84 (−0.27 to 16.14)	−0.06 (−0.16 to 0.04)		IRMS	2012 (Jun–Jul)	[47]
Stem water (Mpala central Kenya)	−2.46 (−4.71 to 0.59)	0.02 (0.01 to 0.03)		IRMS	2012 (Jun–Jul)	[47]
Pond (Sistan Oasis, Iran)	25.83 (13.81 to 29.07)	−144.4 (−172 to –56)		IRMS	–	[54]
Lake and pond	−8.06 (−16.03 to 4.19)	0.02 (−0.02 to 0.04)	0.528	IRMS	2002, 2008	[26]
Surface water	−8.88 (−20.31 to 9.56)	−46.4 to 55.67 (mostly 14 to 33)	0.528	IRMS	Global scale	[36]
River, terminal lakes, well, spring, irrigation channel (Sistan Oasis, Iran)	−1.69 (−6.84 to 14.03)	−3.91 (−59 to 25)	–	IRMS	–	[54]
Lake and river (western USA)	−9.58 (−19.11 to −0.34)	5.28 (−39 to 46)		IRMS		[49]
Dam water (Mpala central Kenya)	−1.08 (−3.98 to 2.26)	0.01 (−0.004 to 0.02)		IRMS	2012 (Jun–Jul)	[47]
Tap, spring water (Israel)	−4.99 (−5.74 to –4.24)	0.04 (0.03 to 0.05)	0.515	IRMS	2008	[26]
Tap water (continental USA)	−8 ± 4.7 (−20.7 to −0.7)	17 ± 11 (−6 to 43)	0.526 to 0.527	IRMS	2006 to 2011	[46]
Tap and puddle water (Mpala central Kenya)	3.36 (2.33 to 4.39)	−0.01 (−0.01 to −0.003)	–	IRMS	2012 (Jun–Jul)	[47]
Precipitation (Northwest Switzerland)	−9.9	18		Picarro CRDS	2012 (Mar) to 2014 (Jun)	[69]
Precipitation (Okinawa Island, Japan)	−4.81 (−9.88 to −1.05)	25.26 (4 to 54)		Picarro CRDS	2011 (Jan) to 2012 (Dec)	[71]
Precipitation (central USA)	−6.25	31	0.5275	OA-ICOS	2014 (Jun) to 2018 (May)	[80]
Rain (Indonesia, India, and Israel)	−5.45 (−9.03 to –2.53)	0.04 (0.02 to 0.06)	0.522	IRMS	2005 (Mar to Nov) 2001 to 2008 2008 (Feb to Aug)	[26]
Water vapor (south Indian and Southern Ocean)	−15.45 (−23.41 to −11.65)	13.51 (−6 to 46)	0.532	IRMS	2005 (Dec) to 2006 (Jan)	[42]
Seawater (Atlantic, Pacific Ocean, Mediterranean, and Northern Red Sea)	0.42 (−0.42 to 2.43)	−0.0045 (−0.01 to 0.0038)	0.528	IRMS	–	[26]
Snow (Mt.Zugspitze, Germany)	(−21.7 to −8.3)	(17 to 62)	–	IRMS		[38]
Snow, ice (Canada, Montenegro)	−17.34 (−26.11 to −5.44)	0.02 (−0.01 to 0.04)	0.529	IRMS	2009, 2010	[26]
**High-latitude precipitation**						
Vapor (NEEM, Greenland)	−41.77 (−44.63 to −38.33)	32.33 (15 to 48)	0.528	IRMS	2008 (Aug)	[48]
Snow precipitation (NEEM, Greenland)	−29.60 (−35.53 to −24.33)	35.67 (23 to 43)		IRMS	2008 (Aug)	[48]
Firn core (NEEM, Greenland)	−32.17 (−38.80 to −26.70)	49.70 (30 to 73)	–	IRMS	2003 (Jan) to 2005 (Aug)	[48]
Snow pit (Vostok)	−57.13 (−61.20 to −51.39)	7.43 (−36 to 42)		IRMS	1949 to 2008	[74]
Surface snow (traverse from Zhongshan station to Dome A)	−34.30 (−58.69 to −17.60)	34.95 (9 to 51)		IRMS	2009 (Dec) to 2010 (Jan)	[45]
Snow (transect from Syowa to Dome Fuji)	−47.26 (−56.69 to −30.20)	24.82 (6.1 to 43.8)	–	IRMS	–	[77]
Ice core (Vostok)	−58 (−50.74 to −62.05)	24.31 (−6 to 54)		IRMS	150ka years	[28]
Surface snow (Vostok, Antarctica)	−41.27 (−28.23 to −51.03)	44.5 (25 to 62)	0.528	IRMS	–	[28]
Snow or ice (Dome F, Antarctica)	−58.42	−0.006	–	IRMS		[26]
Ice core (Talos Dome, Antarctica)	−41.67 to −36.52	–	0.5278	IRMS	9.15 to 33.78 ka age	[76]
Ice core (WAIS, Antarctica)	−35.44 (−42.68 to −31.61)	25.22 (2.9 to 38.46)	–	IRMS	25 ka	[30]
Ice core (Taylor Dome, Antarctica)	−39.56 (−42.92 to −37.36)	14.28 (−1 to 28)	0.5313	IRMS	–	[30]
Ice core (Siple Dome Antarctica)	−31.31 (−36.21 to −24.02)	19 (8 to 27)	–	IRMS		[30]
Snow precipitation (Vostok, Antarctica)	−68.47 to −50.51	−27 to 29	0.5308	IRMS	2000 (Feb–Oct)	[34]
Snow precipitation (Vostok, Antarctica)	−58.5 to −52.6	–	0.5269	IRMS	1999 (Dec) to 2000 (Jun)	[77]
Ice core (Dome C, Antarctica)	−56.48 to −46.639		0.5294	IRMS	7.61–24.8 ka age	[76]
Snow precipitation (Dome C, Antarctica)	−55.58 (−69.63 to –38.89)	18.43 (−11 to 47)	0.5282	IRMS	2010 (Sep–Nov)	[77]
Snow (Dome C, Antarctica)	−54.25 (−61.35 to −48.19)	31.64 (14 to 47)	0.5287	IRMS	2010 (Dec) to 2011 (Dec)	[77]
Snow pit (Dome C, Antarctica)	−51.14 (−55.31 to −46.06)	31.68 (17 to 51)	0.528	IRMS	–	[77]

## References

[1] Gat J.R. (1996). Oxygen and hydrogen isotopes in the hydrologic cycle. Annu. Rev. Earth Planet. Sci..

[2] Dansgaard W. (1964). Stable isotopes in precipitation. Tellus.

[3] Kim S., Han Y., Do Hur S., Yoshimura K., Lee J. (2019). Relating moisture transport to stablewater vapor isotopic variations of ambientwintertime along the western coast of Korea. Atmosphere (Basel).

[4] Petit J.R., Jouzel J., Raynaud D., Barkov N.I., Barnola J.-M., Basile I., Bender M., Chappellaz J., Davisk M., Delaygue G. (1999). Climate and atmospheric history of the past 420,000 years from the Vostok ice core, Antarctica The recent completion of drilling at Vostok station in East. Nature.

[5] Nyamgerel Y., Han Y., Kim S., Hong S.B., Lee J., Hur S. (2020). Do Chronological characteristics for snow accumulation on Styx Glacier in northern Victoria Land, Antarctica. J. Glaciol..

[6] Lee J., Worden J., Noone D., Bowman K., Eldering A., Legrande A., Li J.L.F., Schmidt G., Sodemann H. (2011). Relating tropical ocean clouds to moist processes using water vapor isotope measurements. Atmos. Chem. Phys..

[7] Craig H. (1961). Isotopic Variations in Meteoric Waters. Science.

[8] Kern Z., Hatvani I.G., Czuppon G., Fórizs I., Erdélyi D., Kanduč T., Palcsu L., Vreča P. (2020). Isotopic “altitude” and “continental” effects in modern precipitation across the Adriatic-Pannonian region. Water.

[9] Kong Y., Wang K., Li J., Pang Z. (2019). Stable isotopes of precipitation in China: A consideration of moisture sources. Water.

[10] Bronić I.K., Barešić J., Borković D., Sironić A., Mikelić I.L., Vreča P. (2020). Long-Term isotope records of precipitation in Zagreb, Croatia. Water.

[11] Heydarizad M., Raeisi E., Sorí R., Gimeno L. (2019). Developing meteoric water lines for Iran based on air masses and moisture sources. Water.

[12] Lee J., Feng X., Faiia A.M., Posmentier E.S., Kirchner J.W., Osterhuber R., Taylor S. (2010). Isotopic evolution of a seasonal snowcover and its melt by isotopic exchange between liquid water and ice. Chem. Geol..

[13] Lee J., Feng X., Posmentier E.S., Faiia A.M., Taylor S. (2009). Stable isotopic exchange rate constant between snow and liquid water. Chem. Geol..

[14] Lee J., Feng X., Faiia A., Posmentier E., Osterhuber R., Kirchner J. (2010). Isotopic evolution of snowmelt: A new model incorporating mobile and immobile water. Water Resour. Res..

[15] Kim H., Cho S.H., Lee D., Jung Y.Y., Kim Y.H., Koh D.C., Lee J. (2017). Influence of pre-event water on streamflow in a granitic watershed using hydrograph separation. Environ. Earth Sci..

[16] Galewsky J., Steen-Larsen H.C., Field R.D., Worden J., Risi C., Schneider M. (2016). Stable isotopes in atmospheric water vapor and applications to the hydrologic cycle. Rev. Geophys..

[17] Stenni B., Scarchilli C., Masson-Delmotte V., Schlosser E., Ciardini V., Dreossi G., Grigioni P., Bonazza M., Cagnati A., Karlicek D. (2016). Three-year monitoring of stable isotopes of precipitation at Concordia Station, East Antarctica. Cryosphere.

[18] Johnsen S.J., Dahl-Jensen D., Gundestrup N., Steffensen J.P., Clausen H.B., Miller H., Masson-Delmotte V., Sveinbjörnsdottir A.E., White J. (2001). Oxygen isotope and palaeotemperature records from six Greenland ice-core stations: Camp Century, Dye-3, GRIP, GISP2, Renland and NorthGRIP. J. Quat. Sci..

[19] Masson-Delmotte V., Hou S., Ekaykin A., Jouzel J., Aristarain A., Bernardo R.T., Bromwich D., Cattani O., Delmotte M.M., Falourd S. (2008). A review of antarctic surface snow isotopic composition: Observations, atmospheric circulation, and isotopic modeling. J. Clim..

[20] Goursaud S., Masson-Delmotte V., Favier V., Orsi A., Werner M. (2017). Water stable isotopes spatio-temporal variability in Antarctica in 1960–2013: Observations and simulations from the ECHAM5-wiso atmospheric general circulation model. Clim. Past Discuss..

[21] Opel T., Meyer H., Wetterich S., Laepple T., Dereviagin A., Murton J. (2018). Ice wedges as archives of winter paleoclimate: A review. Permafr. Periglac. Process..

[22] Porter T.J., Opel T. (2020). Recent advances in paleoclimatological studies of Arctic wedge- and pore-ice stable-water isotope records. Permafr. Periglac. Process..

[23] Rosman K.J.R., Taylor P.D.P. (1998). Isotopic compositions of the elements 1997. J. Phys. Chem. Ref. Data.

[24] Giauque W.F., Johnston H.L. (1929). An isotope of oxygen, mass 17, in the Earth’s atmosphere. J. Am. Chem. Soc..

[25] Masson-Delmotte V., Dreyfus G., Braconnot P., Johnsen S., Jouzel J., Kageyama M., Landais A., Loutre M.-F., Nouet J., Parrenin F. (2006). Past temperature reconstructions from deep ice cores: Relevance for future climate change. Clim. Past Discuss..

[26] Luz B., Barkan E. (2010). Variations of ^17^O/^16^O and ^18^O/^16^O in meteoric waters. Geochim. Cosmochim. Acta.

[27] Passey B.H., Levin N.E. (2021). Triple Oxygen Isotopes in Meteoric Waters, Carbonates, and Biological Apatites: Implications for Continental Paleoclimate Reconstruction. Rev. Mineral. Geochem..

[28] Landais A., Barkan E., Luz B. (2008). Record of δ^18^O and ^17^O-excess in ice from Vostok Antarctica during the last 150,000 years. Geophys. Res. Lett..

[29] Barkan E., Luz B. (2007). Diffusivity fractionations of H216O/H 217O and H216O/H218O in air and their implications for isotope hydrology. Rapid Commun. Mass Spectrom..

[30] Schoenemann S.W., Steig E.J., Ding Q., Markle B.R., Schauer (2014). Triple water-isotopologue record from WAIS Divide, Antarctica: Controls on glacial-interglacial changes in ^17^O-excess of precipitation. J. Geophys. Res. Atmos..

[31] Merlivat L., Jouzel J.O. (1979). Global climatic interpretation of the deuterium-oxygen 18 relationship for precipitation. J. Geophys. Res..

[32] Jouzel J., Froehlich K., Schotterer U. (1997). Deutérium et oxygène-18 dans les précipitations contemporaines: Données et modélisation. Hydrol. Sci. J..

[33] Angert A., Cappa C.D., DePaolo D.J. (2004). Kinetic ^17^O effects in the hydrologic cycle: Indirect evidence and implications. Geochim. Cosmochim. Acta.

[34] Landais A., Ekaykin A., Barkan E., Winkler R., Luz B. (2012). Seasonal variations of ^17^O-excess and d-excess in snow precipitation at Vostok station, East Antarctica. J. Glaciol..

[35] Vreča P., Kern Z. (2020). Use of water isotopes in hydrological processes. Water.

[36] Aron P.G., Levin N.E., Beverly E.J., Huth T.E., Passey B.H., Pelletier E.M., Poulsen C.J., Winkelstern I.Z., Yarian D.A. (2020). Triple oxygen isotopes in the water cycle. Chem. Geol..

[37] Tian C., Wang L., Tian F., Zhao S., Jiao W. (2019). O-excess in China. Geochim. Cosmochim. Acta..

[38] Surma J., Assonov S., Staubwasser M. (2021). Triple Oxygen Isotope Systematics in the Hydrologic Cycle. Rev. Mineral. Geochem..

[39] Thiemens M.H., Heidenreich J.E. (1983). The mass-independent fractionation of oxygen: A novel isotope effect and its possible cosmochemical implications. Science.

[40] Barkan E., Luz B. (2005). High precision measurements of and ^18^O/^16^O ratios in H_2_O. Rapid Commun. Mass Spectrom..

[41] Meijer H.A.J., Li W.J. (1998). The Use of Electrolysis for Accurate δO and δO Isotope Measurements in Water. Isot. Environ. Health Stud..

[42] Uemura R., Barkan E., Abe O., Luz B. (2010). Triple isotope composition of oxygen in atmospheric water vapor. Geophys. Res. Lett..

[43] Bao H., Cao X., Hayles J.A. (2016). Triple Oxygen Isotopes: Fundamental Relationships and Applications. Annu. Rev. Earth Planet. Sci..

[44] Schoenemann S.W., Schauer A.J., Steig E.J. (2013). Measurement of SLAP2 and GISP δ17O and proposed VSMOW-SLAP normalization for δ^17^O and ^17^O excess. Rapid Commun. Mass Spectrom..

[45] Pang H., Hou S., Landais A., Masson-Delmotte V., Prie F., Steen-Larsen H.C., Risi C., Li Y., Jouzel J., Wang Y. (2015). Spatial distribution of ^17^O-excess in surface snow along a traverse from Zhongshan station to Dome, East Antarctica. Earth Planet. Sci. Lett..

[46] Li S., Levin N.E., Chesson L.A. (2015). Continental scale variation in ^17^O-excess of meteoric waters in the United States. Geochim. Cosmochim. Acta.

[47] Li S., Levin N.E., Soderberg K., Dennis K.J., Caylor K.K. (2017). Triple oxygen isotope composition of leaf waters in Mpala, central Kenya. Earth Planet. Sci. Lett..

[48] Landais A., Steen-Larsen H.C., Guillevic M., Masson-Delmotte V., Vinther B., Winkler R. (2012). Triple isotopic composition of oxygen in surface snow and water vapor at NEEM (Greenland). Geochim. Cosmochim. Acta..

[49] Passey B.H., Ji H. (2019). Triple oxygen isotope signatures of evaporation in lake waters and carbonates: A case study from the western United States. Earth Planet. Sci. Lett..

[50] Martin F.M. (2002). Isotopic fractionation and the quantification of ^17^O anomalies in the oxygen three-isotope system: An appraisal and geochemical significance. Geochim. Cosmochim. Acta..

[51] McKeegan K.D., Leshin L.A. (2001). Stable isotope variations in extraterrestrial materials. Rev. Mineral. Geochem..

[52] Baker L., Franchi I.A., Maynard J., Wright I.P., Pillinger C.T. (2002). A Technique for the Determination of ^18^O/^16^O and O/^16^O isotopic Ratios in Water from Small Liquid and Solid Samples. Anal. Chem..

[53] Jabeen I., Kusakabe M. (1997). Determination of δ^17^O values of reference water samples VSMOW and SLAP. Chemical Geology.

[54] Surma J., Assonov S., Bolourchi M.J., Staubwasser M. (2015). Triple oxygen isotope signatures in evaporated water bodies from the Sistan Oasis, Iran. Geophys. Res. Lett..

[55] Affek H.P., Barkan E. (2018). A new method for high-precision measurements of ^17^O/^16^O ratios in H_2_O. Rapid Commun. Mass Spectrom..

[56] Barkan E., Musan I., Luz B. (2015). High-precision measurements of δ^17^O and ^17^Oexcess of NBS19 and NBS18. Rapid Commun. Mass Spectrom..

[57] Schauer A.J., Schoenemann S.W., Steig E.J. (2016). Routine high-precision analysis of triple water-isotope ratios using cavity ring-down spectroscopy. Rapid Commun. Mass Spectrom..

[58] Wassenaar L.I., Ahmad M., Aggarwal P., Van Duren M., Pöltenstein L., Araguas L., Kurttas T. (2012). Worldwide proficiency test for routine analysis of δ^2^H and δ^18^O in water by isotope-ratio mass spectrometry and laser absorption spectroscopy. Rapid Commun. Mass Spectrom..

[59] Pierchala A., Rozanski K., Dulinski M., Gorczyca Z., Marzec M., Czub R. (2019). High-precision measurements of δ^2^H, δ^18^O and δ^17^O in water with the aid of cavity ring-down laser spectroscopy. Isot. Environ. Health Stud..

[60] Berman E.S.F., Levin N.E., Landais A., Li S., Owano T. (2013). Measurement of δ^18^O, δ^17^O, and ^17^O-excess in Water by Off-Axis Spectrometry, Integrated Cavity Output Spectroscopy and Isotope Ratio Mass. NIH Public Access.

[61] Steig E.J., Gkinis V., Schauer A.J., Schoenemann S.W., Samek K., Hoffnagle J., Dennis K.J., Tan S.M. (2014). Calibrated high-precision ^17^O-excess measurements using cavity ring-down spectroscopy with laser-current-tuned cavity resonance. Atmos. Meas. Tech..

[62] Sturm P., Knohl A. (2010). Water vapor δ^2^H and δ^18^O measurements using off-axis integrated cavity output spectroscopy. Atmos. Meas. Tech..

[63] Wassenaar L.I., Terzer-Wassmuth S., Douence C., Araguas-Araguas L., Aggarwal P.K., Coplen T.B. (2018). Seeking excellence: An evaluation of 235 international laboratories conducting water isotope analyses by isotope-ratio and laser-absorption spectrometry. Rapid Commun. Mass Spectrom..

[64] Skrzypek G., Ford D. (2014). Stable isotope analysis of saline water samples on a cavity ring-down spectroscopy instrument. Environ. Sci. Technol..

[65] Hendry M.J., Richman B., Wassenaar L.I. (2011). Correcting for methane interferences on δ^2^H and δ^18^O measurements in pore water using H_2_O (liquid)-H_2_O (vapor) equilibration laser spectroscopy. Anal. Chem..

[66] Risi C., Landais A., Winkler R., Vimeux F. (2013). Geoscientific Instrumentation Methods and Data Systems Can we determine what controls the spatio-temporal distribution of d-excess and O-excess in precipitation using the LMDZ general circulation model?. Clim. Past..

[67] Horita J., Wesolowski D.J. (1994). Horita and Wesolowski 1994. Geochim. Cosmochim. Acta..

[68] Tian C., Jiao W., Beysens D., Kaseke K.F., Medici M.-G., Li F., Wang L. (2021). Investigating the role of evaporation in dew formation under different climates using 17O-excess. J. Hydrol..

[69] Affolter S., Häuselmann A.D., Fleitmann D., Häuselmann P., Leuenberger M. (2015). Triple isotope (δD, δ^17^O, δ^18^O) study on precipitation, drip water and speleothem fluid inclusions for a Western Central European cave (NW Switzerland). Quat. Sci. Rev..

[70] Landais A., Risi C., Bony S., Vimeux F., Descroix L., Falourd S., Bouygues A. (2010). Combined measurements of ^17^O excess and d-excess in African monsoon precipitation: Implications for evaluating convective parameterizations. Earth Planet. Sci. Lett..

[71] Uechi Y., Uemura R. (2019). Dominant influence of the humidity in the moisture source region on the ^17^O-excess in precipitation on a subtropical island. Earth Planet. Sci. Lett..

[72] Bershaw J., Hansen D.D., Schauer A.J. (2020). Deuterium excess and 17O-excess variability in meteoric water across the Pacific Northwest, USA. Tellus B Chem. Phys. Meteorol..

[73] Miller M.F. (2018). Precipitation regime influence on oxygen triple-isotope distributions in Antarctic precipitation and ice cores. Earth Planet. Sci. Lett..

[74] Winkler R., Landais A., Risi C., Baroni M., Ekaykin A., Jouzel J., Petit J.R., Prie F., Minster B., Falourd S. (2013). Interannual variation of water isotopologues at Vostok indicates a contribution from stratospheric water vapor. Proc. Natl. Acad. Sci. USA.

[75] Schoenemann S.W., Steig E.J. (2016). Seasonal and spatial variations of ^17^O excess and d-excess in Antarctic precipitation: Insights from an intermediate complexity isotope model. J. Geophys. Res. Atmos..

[76] Winkler R., Landais A., Sodemann H., Dümbgen L., Prié F., Masson-Delmotte V., Stenni B., Jouzel J. (2012). Deglaciation records of ^17^O-excess in East Antarctica: Reliable reconstruction of oceanic normalized relative humidity from coastal sites. Clim. Past..

[77] Touzeau A., Landais A., Stenni B., Uemura R., Fukui K., Fujita S., Guilbaud S., Ekaykin A., Casado M., Barkan E. (2016). Acquisition of isotopic composition for surface snow in East Antarctica and the links to climatic parameters. Cryosphere.

[78] Pang H., Hou S., Landais A., Delmotte V.M., Jouzel J. (2019). Influence of Summer Sublimation on δD, δ^18^O, and δ^17^O in Precipitation, East Antarctica, and Implications for Climate Reconstruction From Ice Cores. J. Geophys. Res. Atmos..

[79] Zahn A., Franz P., Bechtel C., Grooß J.U., Röckmann T. (2006). Modelling the budget of middle atmospheric water vapour isotopes. Atmos. Chem. Phys..

[80] Tian C., Wang L. (2019). Data Descriptor: Stable isotope variations of daily precipitation from 2014–2018 in the central United States. Nat. Publ. Gr..

